# Software Verification: 10th Comparative Evaluation (SV-COMP 2021)

**DOI:** 10.1007/978-3-030-72013-1_24

**Published:** 2021-02-26

**Authors:** Dirk Beyer

**Affiliations:** 8 Eindhoven University of Technology, Eindhoven, The Netherlands; 9 Aalborg University, Aalborg East, Denmark; LMU Munich, Munich, Germany

**Keywords:** Formal Verification, Program Analysis, Competition, Software Verification, Verification Tasks, Benchmark, C Language, Java Language, SV-Benchmarks

## Abstract

SV-COMP 2021 is the 10th edition of the Competition on Software Verification (SV-COMP), which is an annual comparative evaluation of fully automatic software verifiers for C and Java programs. The competition provides a snapshot of the current state of the art in the area, and has a strong focus on reproducibility of its results. The competition was based on 15 201 verification tasks for C programs and 473 verification tasks for Java programs. Each verification task consisted of a program and a property (reachability, memory safety, overflows, termination). SV-COMP 2021 had 30 participating verification systems from 27 teams from 11 countries.

## 1 Introduction

Among several other objectives, the Competition on Software Verification (SV-COMP, `https://sv-comp.sosy-lab.org/2021`) showcases the state of the art in the area of automatic software verification. This edition of SV-COMP is already the 10th edition of the competition and presents again an overview of the currently achieved results by tool implementations that are based on the most recent ideas, concepts, and algorithms for fully automatic verification. This competition report describes the (updated) rules and definitions, presents the competition results, and discusses some interesting facts about the execution of the competition experiments. The objectives of the competitions were discussed earlier (1-4 [16]) and extended over the years (5-6 [17]):

1. provide an overview of the state of the art in software-verification technology and increase visibility of the most recent software verifiers,
2. establish a repository of software-verification tasks that is publicly available for free use as standard benchmark suite for evaluating verification software,

This report extends previous reports on SV-COMP [10, 11, 12, 13, 14, 15, 16, 17].
Reproduction packages are available on Zenodo (see Table 4).
Funded in part by the Deutsche Forschungsgemeinschaft (DFG) – 378803395 (ConVeY).
✉ dirk.beyer@sosy-lab.org

3. establish standards that make it possible to compare different verification tools, including a property language and formats for the results,
4. accelerate the transfer of new verification technology to industrial practice by identifying the strengths of the various verifiers on a diverse set of tasks,
5. educate PhD students and others on performing reproducible benchmarking, packaging tools, and running robust and accurate research experiments, and
6. provide research teams that do not have sufficient computing resources with the opportunity to obtain experimental results on large benchmark sets.

The previous report [17] discusses the outcome of the SV-COMP competition so far with respect to these objectives.

**Related Competitions.** Competitions are an important evaluation method and there are many competitions in the field of formal methods. We refer to the previous report [17] for a more detailed discussion and give here only the references to the most related competitions [9, 19, 55, 56].

**Quick Summary of Changes.** We strive to continuously improve the competition, and this report describes the changes of the last year. In the following we list a brief summary of new items in SV-COMP 2021:

- SPDX identification of licenses in SV-Benchmarks collection
- WitnessLint: New checker for syntactical validity of verification witnesses
- Upgrade of the task-definition format to version 2.0
- Addition of several verification tasks and whole new sub-categories to the SV-Benchmarks collection
- Elimination of competition-specific functions `__VERIFIER_error` and `__VERIFIER_assume` from the verification tasks (and rules)
- Change in scoring schema: Unconfirmed results not counted anymore (when validation was applied)
- CoVeriTeam: New tool that can be used to remotely execute verification runs on the competition machines
- Automatic participation of previous verifiers

## 2 Organization, Definitions, Formats, and Rules

**Procedure.** The overall organization of the competition did not change in comparison to the earlier editions [10, 11, 12, 13, 14, 15, 16, 17]. SV-COMP is an open competition (also known as comparative evaluation), where all verification tasks are known before the submission of the participating verifiers, which is necessary due to the complexity of the C language. The procedure is partitioned into the *benchmark submission* phase, the *training* phase, and the *evaluation* phase. The participants received the results of their verifier continuously via e-mail (for pre-runs and the final competition run), and the results were publicly announced on the competition web site after the teams inspected them. The *Competition Jury* oversees the process and consists of the competition chair and one member of each participating team. Team representatives of the jury are listed in Table 5.

Table 1: Tools for witness-based result validation (validators) and witness linter

| Validator | References | Represent./Developer | Affiliation |
|---|---|---|---|
| CPAchecker | [22, 23, 25] | Martin Spiessl | LMU Munich, Germany |
| UAutomizer | [22, 23] | Daniel Dietsch | Uni Freiburg, Germany |
| CPA-w2t | [24] | Thomas Lemberger | LMU Munich, Germany |
| FShell-w2t | [24] | Michael Tautschnig | Queen Mary U. of London, UK |
| NITWIT | [78] | Philipp Berger | RWTH Aachen, Germany |
| MetaVal | [29] | Martin Spiessl | LMU Munich, Germany |
| WitnessLint | | Sven Umbricht | LMU Munich, Germany |

**License Requirements.** Starting 2018, SV-COMP required that the verifier must be publicly available for download and has a license that

(i) allows reproduction and evaluation by anybody (incl. results publication),
(ii) does not restrict the usage of the verifier output (log files, witnesses), and
(iii) allows any kind of (re-)distribution of the unmodified verifier archive.

During the qualification phase, when the jury members inspect the verifier archives, several issues with licenses (missing licenses, incompatibilities) were detected that the developers were able to address the issues on time.

With SV-COMP 2021, the community started the process of making the benchmark collection REUSE compliant (`https://reuse.software`) by adding SPDX license identifiers (`https://spdx.dev`). A few directories are properly labeled already, and continuous-integration checks with REUSE ensure that new contributions adhere to the standard.

**Validation of Results.** This time, the validation of the verification results was done by seven validation tools, which are listed in Table 1, including references to literature. The validators CPAchecker and UAutomizer support the competition since the beginning of its result validation in 2015. Execution-based validation was added in 2018 using CPA-w2t and FShell-w2t. Two new validators participated since the previous SV-COMP in 2020: Nitwit and MetaVal. A few categories were still excluded from validation because no validators were available for some types of programs or properties.

For SV-COMP 2021, the new validator WitnessLint was added for validating witnesses regarding their syntax. It checks the witnesses produced by the verification tools against the specification of the format for verification witnesses (`https://github.com/sosy-lab/sv-witnesses/tree/svcomp21`). For example, WitnessLint ensures that a verification witness is a proper XML/GraphML file and contains the required meta data. This means that the validators can focus on the validation of the verification result, assuming that the verification witness is syntactically valid. If the witness linter deems a verification witness as syntactically invalid, then the answers of the result validators are ignored and the result is not counted as confirmed.

**Task-Definition Format 2.0.** The format for the task definitions in the SV-Benchmarks repository was recently extended to include a set of options that can carry information from the verification task to the verification tool. SV-COMP 2021 used the task-definition format in version 2.0 (https://gitlab.com/sosy-lab/benchmarking/task-definition-format/-/tree/2.0). More details can be found in the report for Test-Comp 2021 [19].

**Properties.** Please see the 2015 competition report [13] for the definition of the properties and the property format. All specifications are available in the directory `c/properties/` of the benchmark repository.

**Categories.** The updated category structure is illustrated by Fig. 1. The categories are also listed in Tables 7 and 8, and described in detail on the competition web site (https://sv-comp.sosy-lab.org/2021/benchmarks.php). Compared to the category structure for SV-COMP 2020, we added the sub-categories *XCSP* and *Combinations* to category *ReachSafety*, and the sub-categories *DeviceDriversLinux64Large ReachSafety*, *uthash MemSafety*, *uthash NoOverflows*, and *uthash ReachSafety* to category *SoftwareSystems*.

Another effort was to integrate some of the Juliet benchmark tasks [31] into the SV-Benchmarks collection. We requested a license for the Juliet programs that properly clarifies the license terms also outside the USA. We thank our colleagues from NIST for releasing their Juliet benchmark (which is declared as public domain) under the Creative Commons license CC0-1.0 (https://github.com/sosy-lab/sv-benchmarks/blob/svcomp21/LICENSES/CC0-1.0.txt). SV-COMP 2021 used many verification tasks from Juliet, in particular for the memory-safety properties CWE121 (stack-based buffer overflow), CWE401 (memory leak), CWE415 (double free), CWE476 (null-pointer dereference), and CWE590 (free memory that is not on the heap) (see https://github.com/sosy-lab/sv-benchmarks/blob/svcomp21/c/MemSafety-Juliet.set).

All those new contributions to the benchmark collection lead to the growth of the number of verification tasks from 11 052 in SV-COMP 2020 to 15 201 in SV-COMP 2021.

**Verification Tasks.** The previous verification tasks and competition rules used special definitions for the functions `__VERIFIER_error` and `__VERIFIER_assume`. These special definitions were found to be unintuitive and inconsistent with expectations in the verification community, and repeatedly caused confusion among participants. A call of function `__VERIFIER_error()` was defined to never return. A call of function `__VERIFIER_assume(p)` was defined such that if expression $p$ evaluates to false, then the function loops forever, otherwise the function returns without any side effects. This led to unintended interactions with other properties.

We eliminated these two functions in two steps. In the first step, each function call was replaced by a C-code implementation of the intended behavior. In most of the cases, `__VERIFIER_error();` was replaced by the C code `reach_error(); abort();`, where `reach_error` is a 'normal' function, i.e., one whose interpretation follows the C standard [3].

Eliminating `__VERIFIER_assume` was more complicated: In some tasks for property *memory-cleanup*, `__VERIFIER_assume(p);` was replaced by the C code `assume_cycle_if_not(p);`, which is implemented

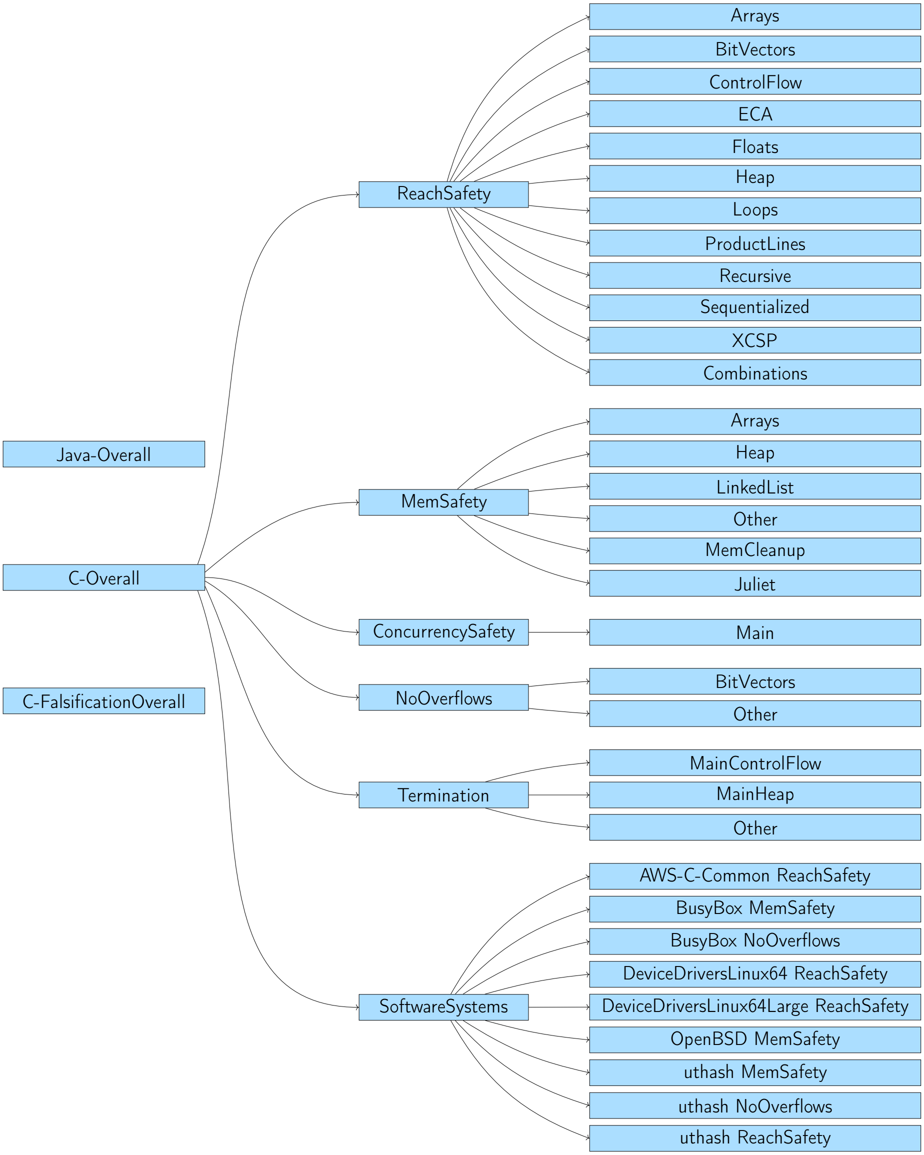

Fig. 1: Category structure for SV-COMP 2021; category *C-FalsificationOverall* contains all verification tasks of *C-Overall* without *Termination*; *Java-Overall* contains all Java verification tasks; compared to SV-COMP 2020, there are two new sub-categories in *ReachSafety* and four new sub-categories in *SoftwareSystems*

Table 2: Scoring schema for SV-COMP 2021 (new: no point for unconfirmed correct results anymore)

| Reported result | Points | Description |
|---|---|---|
| UNKNOWN | 0 | Failure to compute verification result |
| FALSE correct | +1 | Violation of property in program was correctly found and a validator confirmed the result based on a witness |
| FALSE incorrect | −16 | Violation reported but property holds (false alarm) |
| TRUE correct | +2 | Program correctly reported to satisfy property and a validator confirmed the result based on a witness |
| TRUE incorrect | −32 | Incorrect program reported as correct (wrong proof) |

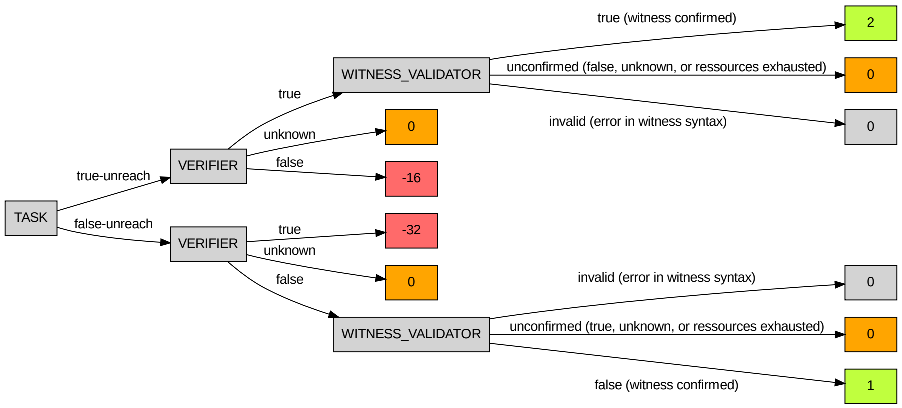

Fig. 2: Visualization of the scoring schema for the reachability property (adjusted from a previous report [15])

as `if (!p) while(1);`, while for other tasks, `__VERIFIER_assume(p);` was replaced by `assume_abort_if_not(p);`, which is implemented as `if (!p) abort();`. The solution nicely illustrates the problem of the special semantics: Consider property *memory-cleanup*, which requires that all allocated memory is deallocated before the program terminates. Here, the desired behavior of a failing assume statement would be that the program does not terminate (and does not unintendedly violate the *memory-cleanup* property). Now consider property *termination*, which requires that every path finally reaches the end of the program. Here, the desired behavior of a failing assume statement would be that the program terminates (and does not unintendedly violate the *termination* property).

In the second step, the specifications for functions `__VERIFIER_error` and `__VERIFIER_assume` were removed from the competition rules (because no such functions exist anymore in the SV-Benchmarks collection).

**Scoring Schema and Ranking.** Table 2 provides an overview and Fig. 2 visually illustrates the score assignment for the reachability property as an example.

The scoring schema was changed regarding the special rule for unconfirmed correct results for expected result True. There was a rule during the transitioning phase to assign one point if the answer matches the expected result but the witness was not confirmed. Now score points are only assigned if the results got validated (or no validator was available).

As in the last years, the rank of a verifier was decided based on the sum of points (normalized for meta categories). In case of a tie, the rank was decided based on success run time, which is the total CPU time over all verification tasks for which the verifier reported a correct verification result. *Opt-out from Categories* and *Score Normalization for Meta Categories* was done as described previously [11] (page 597).

## 3 Reproducibility

To allow independent reproduction of the SV-COMP results, we made all major components that were used in the competition available in public version-control repositories. An overview of the components that contribute to the reproducible setup of SV-COMP is provided in Fig. 3, and the details are given in Table 3. We refer to the SV-COMP 2016 report [14] for a description of all components of the SV-COMP organization.

We have published the competition artifacts at Zenodo (see Table 4) to guarantee their long-term availability and immutability. These artifacts comprise the verification tasks, the competition results, the produced verification witnesses, and the BenchExec package. The archive for the competition results includes the raw results in BenchExec's XML exchange format, the log output of the verifiers and validators, and a mapping from file names to SHA-256 hashes. The hashes of the files are useful for validating the exact contents of a file, and accessing the files inside the archive that contains the verification witnesses.

**Competition Workflow.** The workflow of the competition is described in the report for Test-Comp 2021 [19].

**CoVeriTeam.** The competition was for the first time supported by CoVeriTeam [26] (`https://gitlab.com/sosy-lab/software/coveriteam/`), which is a tool for cooperative verification. Among its many capabilities, it enables remote execution of verification runs directly on the competition machines, which was found to be a valuable service for trouble shooting.

## 4 Results and Discussion

The results of the competition experiments represent the state of the art in fully automatic software-verification tools. The report shows the results, in terms of effectiveness (number of verification tasks that can be solved and correctness of the results, as accumulated in the score) and efficiency (resource consumption in terms of CPU time and CPU energy). The results are presented in the same way as in last years, such that the improvements compared to last year are easy

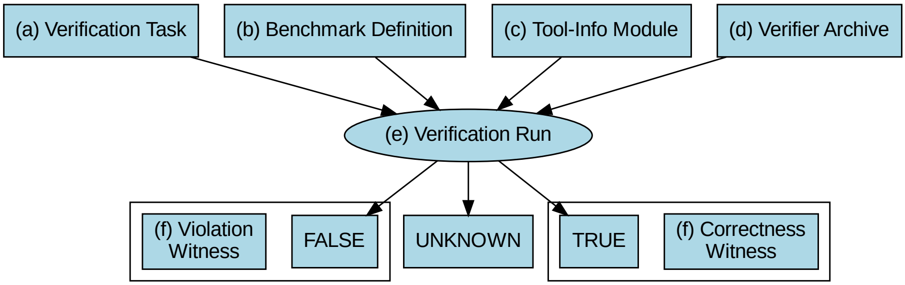

Fig. 3: Benchmarking components of SV-COMP and competition's execution flow (same as for SV-COMP 2020)

Table 3: Publicly available components for reproducing SV-COMP 2021

| Component | Fig. 3 | Repository | Version |
|---|---|---|---|
| Verification Tasks | (a) | github.com/sosy-lab/sv-benchmarks | svcomp21 |
| Benchmark Definitions | (b) | gitlab.com/sosy-lab/sv-comp/bench-defs | svcomp21 |
| Tool-Info Modules | (c) | github.com/sosy-lab/benchexec | 3.6 |
| Verifier Archives | (d) | gitlab.com/sosy-lab/sv-comp/archives-2021 | svcomp21 |
| Benchmarking | (e) | github.com/sosy-lab/benchexec | 3.6 |
| Witness Format | (f) | github.com/sosy-lab/sv-witnesses | svcomp21 |

Table 4: Artifacts published for SV-COMP 2021

| Content | DOI | Reference |
|---|---|---|
| Verification Tasks | 10.5281/zenodo.4459126 | [20] |
| Competition Results | 10.5281/zenodo.4458215 | [18] |
| Verification Witnesses | 10.5281/zenodo.4459196 | [21] |
| BenchExec | 10.5281/zenodo.4317433 | [82] |

to identify. The results presented in this report were inspected and approved by the participating teams. We now discuss the highlights of the results.

**Participating Verifiers.** Table 5 provides an overview of the participating verification systems (see also the listing on the competition web site at https://sv-comp.sosy-lab.org/2021/systems.php). Table 6 lists the algorithms and techniques that are used by the verification tools.

**Automatic Participation.** To ensure that the comparative evaluation continues to give an overview of the state of the art that is as broad as possible, a rule was introduced before SV-COMP 2020 which enables the option for the organizer to reuse systems that participated in previous years for the comparative evaluation. This option was used three times in SV-COMP 2021: for Coastal, PredatorHP, and SPF. Those participations are marked as 'hors concours' in Table 5.

Table 5: Competition candidates with tool references and representing jury members

| Participant | Ref. | Jury member | Affiliation |
|---|---|---|---|
| 2LS | [32, 63] | Viktor Malík | BUT, Brno, Czechia |
| Brick | | Lei Bu | Nanjing U., China |
| Cbmc | [60] | Michael Tautschnig | Queen Mary U. of London, UK |
| Coastal | [79] | (hors concours) | – |
| CPA-BAM-BnB | [4, 81] | Vadim Mutilin | ISP RAS, Russia |
| CPALockator | [5, 6] | Pavel Andrianov | ISP RAS, Russia |
| CPAchecker | [27, 41] | Stephan Holzner | LMU Munich, Germany |
| Dartagnan | [48, 68] | Hernán Ponce de León | U. Bundeswehr Munich, Germany |
| Divine | [8, 61] | Henrich Lauko | Masaryk U., Brno, Czechia |
| ESBMC-incr | [36, 39] | Felipe R. Monteiro | Amazon Web Services, USA |
| ESBMC-kind | [46, 47] | Lucas Cordeiro | U. of Manchester, UK |
| Frama-C | [40] | Martin Spiessl | LMU Munich, Germany |
| Gazer-Theta | [1, 74] | Ákos Hajdu | BME, Hungary |
| Goblint | [73, 80] | Simmo Saan | U. of Tartu, Estonia |
| Java Ranger | [76, 77] | Soha Hussein | U. of Minnesota, USA |
| JayHorn | [59, 75] | Hossein Hojjat | U. of Tehran, Iran |
| Jbmc | [37, 38] | Peter Schrammel | U. of Sussex / Diffblue, UK |
| JDart | [62, 64] | Falk Howar | TU Dortmund, Germany |
| Korn | [45] | Gidon Ernst | LMU Munich, Germany |
| Lazy-CSeq | [57, 58] | Omar Inverso | Gran Sasso Science Institute, Italy |
| PeSCo | [71, 72] | Cedric Richter | Paderborn U., Germany |
| Pinaka | [35] | Saurabh Joshi | IIT Hyderabad, India |
| PredatorHP | [54, 67] | (hors concours) | – |
| Smack | [51, 70] | Zvonimir Rakamaric | U. of Utah, USA |
| SPF | [65, 69] | (hors concours) | – |
| Symbiotic | [33, 34] | Marek Chalupa | Masaryk U., Brno, Czechia |
| UAutomizer | [52, 53] | Matthias Heizmann | U. of Freiburg, Germany |
| UKojak | [44, 66] | Dominik Klumpp | U. of Freiburg, Germany |
| UTaipan | [43, 49] | Daniel Dietsch | U. of Freiburg, Germany |
| VeriAbs | [2, 42] | Priyanka Darke | Tata Consultancy Services, India |

**Computing Resources.** The resource limits were the same as in the previous competitions [14]: Each verification run was limited to 8 processing units (cores), 15 GB of memory, and 15 min of CPU time. Witness validation was limited to 2 processing units, 7 GB of memory, and 1.5 min of CPU time for violation witnesses and 15 min of CPU time for correctness witnesses. The machines for running the experiments are part of a compute cluster that consists of 168 machines; each verification run was executed on an otherwise completely unloaded, dedicated machine, in order to achieve precise measurements. Each machine had one Intel Xeon E3-1230 v5 CPU, with 8 processing units each, a frequency of 3.4 GHz, 33 GB of RAM, and a GNU/Linux operating system (x86_64-linux, Ubuntu 20.04 with Linux kernel 5.4). We used BenchExec [28] to measure and control computing resources (CPU time, memory, CPU energy) and VerifierCloud (`https://vcloud.sosy-lab.org`) to distribute, install, run, and clean-up verification runs, and to collect the results. The values for time and

Table 6: Algorithms and techniques that the competition candidates used

| Participant | CEGAR | Predicate Abstraction | Symbolic Execution | Bounded Model Checking | k-Induction | Property-Directed Reach. | Explicit-Value Analysis | Numeric. Interval Analysis | Shape Analysis | Separation Logic | Bit-Precise Analysis | ARG-Based Analysis | Lazy Abstraction | Interpolation | Automata-Based Analysis | Concurrency Support | Ranking Functions | Evolutionary Algorithms | Algorithm Selection | Portfolio |
|---|---|---|---|---|---|---|---|---|---|---|---|---|---|---|---|---|---|---|---|---|
| 2LS | | | | ✓ | ✓ | | | ✓ | ✓ | | ✓ | | | | | | ✓ | | | |
| Brick | ✓ | | ✓ | ✓ | | | | ✓ | | | | | | | | ✓ | | | | |
| Cbmc | | | | ✓ | | | | | | | ✓ | | | | | ✓ | | | | |
| Coastal | | | ✓ | | | | | | | | | | | | | | | | | |
| CPA-BAM-BnB | ✓ | ✓ | | | | | ✓ | | | | ✓ | ✓ | ✓ | ✓ | | | | | | |
| CPALockator | ✓ | ✓ | | | | | ✓ | | | | ✓ | ✓ | ✓ | ✓ | | ✓ | | | | |
| CPAchecker | ✓ | ✓ | | ✓ | ✓ | | ✓ | ✓ | ✓ | | ✓ | ✓ | ✓ | ✓ | | ✓ | ✓ | | ✓ | ✓ |
| Dartagnan | | | | ✓ | | | | | | | ✓ | | | | | ✓ | | | | |
| Divine | | | ✓ | | | | ✓ | | | | ✓ | | | | | ✓ | | | ✓ | ✓ |
| ESBMC-incr | | | | ✓ | ✓ | | | | | | ✓ | | | | | ✓ | | | | |
| ESBMC-kind | | | | ✓ | ✓ | | | ✓ | | | ✓ | | | | | ✓ | | | | |
| Frama-C | | | | | | | | ✓ | | | | | | | | | | | | |
| Gazer-Theta | ✓ | ✓ | | ✓ | | | ✓ | | | | ✓ | ✓ | ✓ | ✓ | | | | | | ✓ |
| Goblint | | | | | | | | ✓ | | | | | | | | ✓ | | | | |
| Java Ranger | | | ✓ | | | | | | | | ✓ | | | | | | | | | |
| JayHorn | ✓ | ✓ | | | | ✓ | | ✓ | | | | | ✓ | ✓ | | | | | | |
| Jbmc | | | | ✓ | | | | | | | ✓ | | | | | ✓ | | | | |
| JDart | | | ✓ | | | | | | | | ✓ | | | | | | | | | ✓ |
| Korn | | ✓ | ✓ | | | | ✓ | | | | | | | | | | | | | ✓ |
| Lazy-CSeq | | | | ✓ | | | | | | | ✓ | | | | | ✓ | | | | |
| PeSCo | ✓ | ✓ | | ✓ | ✓ | | ✓ | ✓ | ✓ | | ✓ | ✓ | ✓ | ✓ | | ✓ | ✓ | | ✓ | ✓ |
| Pinaka | | | ✓ | ✓ | | | | | | | ✓ | | | | | | | | | |
| PredatorHP | | | | | | | | | ✓ | | | | | | | | | | | |
| Smack | | | | ✓ | | | | | | | ✓ | | ✓ | | | ✓ | | | | |
| SPF | | | ✓ | | | | | | ✓ | | | | | | | ✓ | | | | |
| Symbiotic | | | ✓ | | ✓ | | | ✓ | ✓ | | ✓ | | | | | | | | | ✓ |
| UAutomizer | ✓ | ✓ | | | | | | | | | ✓ | | ✓ | ✓ | ✓ | ✓ | ✓ | | ✓ | ✓ |
| UKojak | ✓ | ✓ | | | | | | | | | ✓ | | ✓ | ✓ | | | | | | |
| UTaipan | ✓ | ✓ | | | | | ✓ | ✓ | | | ✓ | | ✓ | ✓ | ✓ | ✓ | | | ✓ | ✓ |
| VeriAbs | ✓ | | | ✓ | ✓ | | ✓ | ✓ | | | | | | | | | | ✓ | ✓ | ✓ |

energy are accumulated over all cores of the CPU. To measure the CPU energy, we used CPU Energy Meter [30] (integrated in BenchExec [28]).

One complete verification execution of the competition consisted of 163 177 verification runs (each verifier on each verification task of the selected categories according to the opt-outs), consuming 470 days of CPU time and 126 kWh of CPU energy (without validation). Witness-based result validation required 961 919 validation runs (each validator on each verification task for categories with witness validation, and for each verifier), consuming 274 days of CPU time. Each tool was executed several times, in order to make sure no installation issues occur during the execution. Including preruns, the infrastructure managed a total of 1.33 million verification runs consuming 4.16 years of CPU time, and 7.31 million validation runs consuming 3.84 years of CPU time.

**Quantitative Results.** Table 7 presents the quantitative overview of all tools and all categories. The head row mentions the category, the maximal score for the category, and the number of verification tasks. The tools are listed in alphabetical order; every table row lists the scores of one verifier. We indicate the top three candidates by formatting their scores in bold face and in larger font size. An empty table cell means that the verifier opted-out from the respective main category (perhaps participating in subcategories only, restricting the evaluation to a specific topic). More information (including interactive tables, quantile plots for every category, and also the raw data in XML format) is available on the competition web site (`https://sv-comp.sosy-lab.org/2021/results`) and in the results artifact (see Table 4).

Table 8 reports the top three verifiers for each category. The run time (column 'CPU Time') and energy (column 'CPU Energy') refer to successfully solved verification tasks (column 'Solved Tasks'). We also report the number of tasks for which no witness validator was able to confirm the result (column 'Unconf. Tasks'). The columns 'False Alarms' and 'Wrong Proofs' report the number of verification tasks for which the verifier reported wrong results, i.e., reporting a counterexample when the property holds (incorrect False) and claiming that the program fulfills the property although it actually contains a bug (incorrect True), respectively.

**Score-Based Quantile Functions for Quality Assessment.** We use score-based quantile functions [11, 28] because these visualizations make it easier to understand the results of the comparative evaluation. The web site (`https://sv-comp.sosy-lab.org/2021/results`) and the results archive (see Table 4) include such a plot for each (sub-)category. As an example, we show the plot for category *C-Overall* (all verification tasks) in Fig. 4. A total of 10 verifiers participated in category *C-Overall*, for which the quantile plot shows the overall performance over all categories (scores for meta categories are normalized [11]). A more detailed discussion of score-based quantile plots, including examples of what insights one can obtain from the plots, is provided in previous competition reports [11, 14].

**Alternative Rankings.** The community suggested to report a couple of alternative rankings that honor different aspects of the verification process as complement to the official SV-COMP ranking. Table 9 is similar to Table 8, but

Table 7: Quantitative overview over all results; empty cells represent opt-outs; an asterisk after the tool name marks hors-concours participation

| **Participant** | **ReachSafety** 7844 points 4927 tasks | **MemSafety** 4981 points 3296 tasks | **ConcurrencySafety** 1413 points 1130 tasks | **NoOverflows** 682 points 452 tasks | **Termination** 3897 points 2212 tasks | **SoftwareSystems** 5608 points 3184 tasks | **FalsificationOverall** 6173 points 12989 tasks | **Overall** 23778 points 15201 tasks | **JavaOverall** 693 points 473 tasks |
|---|---|---|---|---|---|---|---|---|---|
| 2ls | 3021 | 1100 | 0 | 414 | **1315** | -7 | 1436 | 6219 | |
| Brick | | | | | | | | | |
| Cbmc | 3395 | -725 | 486 | 279 | 872 | 565 | 2609 | 5289 | |
| CPA-BAM-BnB | | | | | | 491 | | | |
| CPALockator | | | -819 | | | | | | |
| CPAchecker | **4764** | **2992** | **1050** | **531** | **1356** | 736 | **4356** | **12217** | |
| Dartagnan | | | 309 | | | | | | |
| Divine | 2012 | 95 | 391 | 0 | 0 | 124 | 306 | 2083 | |
| ESBMC-incr | | | -134 | | | | | | |
| ESBMC-kind | 4486 | 1281 | 37 | 317 | 832 | 694 | 2002 | 6656 | |
| Frama-C | | | | 172 | | | | | |
| Gazer-Theta | | | | | | | | | |
| Goblint | 777 | | 46 | 156 | | 331 | | | |
| Korn | | | | | | | | | |
| Lazy-CSeq | | | **1206** | | | | | | |
| PeSCo | **4526** | | | | | **878** | **4329** | **12208** | |
| Pinaka | 3408 | | | -200 | 669 | | | | |
| PredatorHP* | | 2187 | | | | | | | |
| Smack | | | | | | **894** | | | |
| Symbiotic | 3864 | **3125** | 0 | 373 | 1043 | **2001** | 2947 | 9268 | |
| UAutomizer | 3502 | **1615** | **943** | **512** | **3019** | 359 | **3432** | **11769** | |
| UKojak | 1768 | 925 | 0 | 441 | 0 | 298 | 1800 | 4332 | |
| UTaipan | 2743 | 1436 | 937 | **506** | 0 | 282 | 3336 | 7676 | |
| VeriAbs | **5771** | | | | | | | | |
| Coastal* | | | | | | | | | 298 |
| Java Ranger | | | | | | | | | **630** |
| JayHorn | | | | | | | | | 369 |
| Jbmc | | | | | | | | | **603** |
| JDart | | | | | | | | | **623** |
| SPF* | | | | | | | | | 409 |

Table 8: Overview of the top-three verifiers for each category (measurement values for CPU time and energy rounded to two significant digits)

| Rank | Verifier | Score | CPU Time (in h) | CPU Energy (in kWh) | Solved Tasks | Unconf. Tasks | False Alarms | Wrong Proofs |
|---|---|---|---|---|---|---|---|---|
| ***ReachSafety*** | | | | | | | | |
| 1 | **VeriAbs** | **5771** | 130 | 1.5 | 3 526 | 725 | | |
| 2 | CPAchecker | 4764 | 100 | 1.2 | 2 922 | 251 | 6 | |
| 3 | PeSCo | 4526 | 53 | 0.48 | 2 820 | 272 | 7 | |
| ***MemSafety*** | | | | | | | | |
| 1 | **Symbiotic** | **3125** | 1.6 | 0.021 | 370 | 8 | | |
| 2 | CPAchecker | 2992 | 7.8 | 0.069 | 3 092 | 0 | | |
| 3 | UAutomizer | 1615 | 4.1 | 0.046 | 160 | 2 | | |
| ***ConcurrencySafety*** | | | | | | | | |
| 1 | **Lazy-CSeq** | **1206** | 4.0 | 0.051 | 985 | 34 | | |
| 2 | CPAchecker | 1050 | 16 | 0.13 | 903 | 0 | 1 | |
| 3 | UAutomizer | 943 | 9.6 | 0.087 | 775 | 176 | | |
| ***NoOverflows*** | | | | | | | | |
| 1 | **CPAchecker** | **531** | 1.2 | 0.012 | 366 | 3 | | |
| 2 | UAutomizer | 512 | 1.7 | 0.015 | 358 | 0 | | |
| 3 | UTaipan | 506 | 1.9 | 0.018 | 355 | 0 | | |
| ***Termination*** | | | | | | | | |
| 1 | **UAutomizer** | **3019** | 22 | 0.24 | 1 581 | 9 | | |
| 2 | CPAchecker | 1356 | 17 | 0.20 | 1 078 | 70 | 10 | |
| 3 | 2ls | 1315 | 2.5 | 0.021 | 977 | 363 | 3 | |
| ***SoftwareSystems*** | | | | | | | | |
| 1 | **Symbiotic** | **2001** | 0.55 | 0.0075 | 1 024 | 128 | | |
| 2 | Smack | 894 | 14 | 0.14 | 1 362 | 58 | | **2** |
| 3 | PeSCo | 878 | 27 | 0.27 | 1 484 | 234 | 1 | |
| ***FalsificationOverall*** | | | | | | | | |
| 1 | **CPAchecker** | **4356** | 71 | 0.76 | 3 814 | 98 | 8 | |
| 2 | PeSCo | 4329 | 47 | 0.41 | 3 798 | 106 | 9 | |
| 3 | UAutomizer | 3432 | 30 | 0.30 | 1 585 | 215 | 1 | |
| ***Overall*** | | | | | | | | |
| 1 | **CPAchecker** | **12217** | 190 | 2.1 | 9 835 | 514 | 18 | |
| 2 | PeSCo | 12208 | 120 | 1.2 | 9 743 | 579 | 19 | |
| 3 | UAutomizer | 11769 | 99 | 1.0 | 5 980 | 489 | 1 | **1** |
| ***JavaOverall*** | | | | | | | | |
| 1 | **Java Ranger** | **630** | 4.9 | 0.056 | 427 | 0 | | |
| 2 | JDart | 623 | 0.93 | 0.0093 | 437 | 0 | | |
| 3 | Jbmc | 603 | 0.22 | 0.0022 | 423 | 0 | | |

contains the alternative ranking categories *Correct* and *Green Verifiers*. Column 'Quality' gives the score in score points, column 'CPU Time' the CPU usage of successful runs in hours, column 'CPU Energy' the CPU usage of successful runs in kWh, column 'Solved Tasks' the number of correct results, column 'Wrong Re-

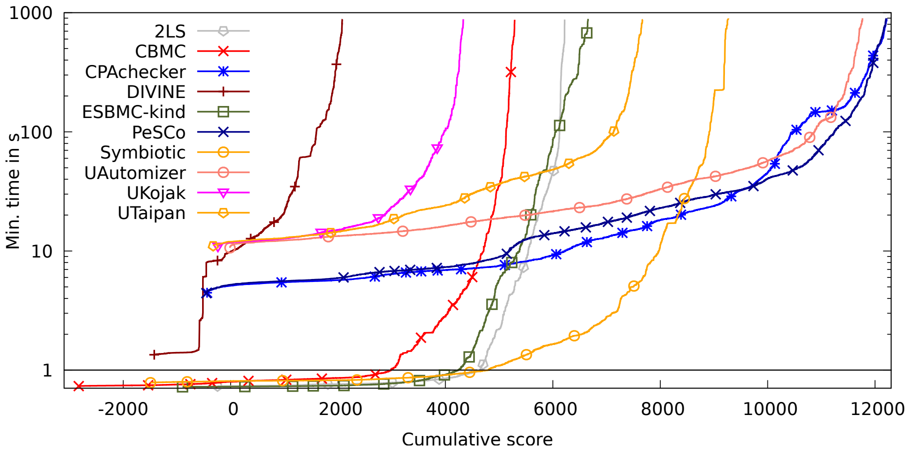

Fig. 4: Quantile functions for category *C-Overall*. Each quantile function illustrates the quantile ($x$-coordinate) of the scores obtained by correct verification runs below a certain run time ($y$-coordinate). More details were given previously [11]. A logarithmic scale is used for the time range from 1 s to 1000 s, and a linear scale is used for the time range between 0 s and 1 s.

Table 9: Alternative rankings for catagory *Overall*; quality is given in score points (sp), CPU time in hours (h), kilo-watt-hours (kWh), wrong results in errors (E), rank measures in errors per score point (E/sp), joule per score point (J/sp), and score points (sp)

| **Rank** | **Verifier** | **Quality (sp)** | **CPU Time (h)** | **CPU Energy (kWh)** | **Solved Tasks** | **Wrong Results (E)** | **Rank Measure** |
|---|---|---|---|---|---|---|---|
| ***Correct Verifiers*** | | | | | | | (E/sp) |
| 1 | **UAutomizer** | 11 769 | 99 | 1.0 | 5 980 | 2 | .00017 |
| 2 | UKojak | 4 332 | 46 | 0.48 | 2 476 | 1 | .00023 |
| 3 | CPAchecker | 12 217 | 190 | 2.1 | 9 835 | 18 | .0015 |
| worst | | | | | | 48 | .023 |
| ***Green Verifiers*** | | | | | | | (J/sp) |
| 1 | **Symbiotic** | 9 268 | 21 | 0.26 | 4 999 | 16 | 100 |
| 2 | 2ls | 6 219 | 26 | 0.24 | 3 372 | 12 | 140 |
| 3 | Cbmc | 5 289 | 26 | 0.31 | 5 596 | 52 | 210 |
| worst | | | | | | | 630 |

sults' the sum of false alarms and wrong proofs in number of errors, and column 'Rank Measure' gives the measure to determine the alternative rank.

*Correct Verifiers — Low Failure Rate.* The right-most columns of Table 8 report that the verifiers achieve a high degree of correctness (all top three verifiers in the *C-Overall* have less than 2 ‰ wrong results). The winners of category *Java-Overall* produced not a single wrong answer. The first category in

Table 10: New verifiers in SV-COMP 2020 and SV-COMP 2021

| Verifier | Language | First Year | Sub-categories |
|---|---|---|---|
| Frama-C | C | 2021 | 4 |
| Gazer-Theta | C | 2021 | 9 |
| Goblint | C | 2021 | 25 |
| Korn | C | 2021 | 13 |
| Brick | C | 2020 | 1 |
| Dartagnan | C | 2020 | 5 |
| Gacal | C | 2020 | 1 |
| Coastal | Java | 2020 | 1 |
| Java Ranger | Java | 2020 | 1 |
| JDart | Java | 2020 | 1 |

Table 11: Confirmation rate of verification witnesses in SV-COMP 2021

| Result | True | | | | False | | | |
|---|---|---|---|---|---|---|---|---|
| | Total | Confirmed | | Unconf. | Total | Confirmed | | Unconf. |
| **2ls** | 2 252 | 2 245 | **99.7 %** | **7** | 1 591 | 1 127 | 70.8 % | 464 |
| Cbmc | 3 875 | 3 498 | 90.3 % | 377 | 3 772 | 2 098 | 55.6 % | 1 674 |
| CPAchecker | 5 992 | 5 646 | 94.2 % | 346 | 4 357 | 4 189 | 96.1 % | 168 |
| Divine | 1 673 | 1 649 | 98.6 % | 24 | 1 317 | 986 | 74.9 % | 331 |
| ESBMC-kind | 4 954 | 4 901 | 98.9 % | 53 | 1 736 | 1 625 | 93.6 % | 111 |
| PeSCo | 5 973 | 5 570 | 93.3 % | 403 | 4 349 | 4 173 | 96.0 % | 176 |
| Symbiotic | 3 351 | 3 149 | 94.0 % | 202 | 2 166 | 1 850 | 85.4 % | 316 |
| UAutomizer | 4 121 | 3 856 | 93.6 % | 265 | 2 348 | 2 124 | 90.5 % | 224 |
| **UKojak** | 1 816 | 1 796 | 98.9 % | 20 | 690 | 680 | **98.6 %** | **10** |
| UTaipan | 2 602 | 2 542 | 97.7 % | 60 | 1 637 | 1 417 | 86.6 % | 220 |

Table 9 uses a failure rate as rank measure: $\frac{\textsf{number of incorrect results}}{\textsf{total score}}$, the number of errors per score point ($E/sp$). We use $E$ as unit for number of incorrect results and $sp$ as unit for total score. The worst result was 0.032 E/sp in SV-COMP 2020 and is now improved to 0.023 E/sp.

*Green Verifiers — Low Energy Consumption.* Since a large part of the cost of verification is given by the energy consumption, it might be important to also consider the energy efficiency. The second category in Table 9 uses the energy consumption per score point as rank measure: $\frac{\textsf{total CPU energy}}{\textsf{total score}}$, with the unit $J/sp$. The worst result from SV-COMP 2020 was 2 200 J/sp, now improved to 630 J/sp.

*New Verifiers.* To acknowledge the verification systems that participate for the first or second time in SV-COMP, Table 10 lists the new verifiers (in SV-COMP 2020 or SV-COMP 2021).

**Verifiable Witnesses.** Results validation is of primary importance in the competition. All SV-COMP verifiers are required to justify the result (True or False) by producing a verification witness (except for those categories for which no witness validator is available). We used six independently developed witness-based result validators and one witness linter (see Table 1).

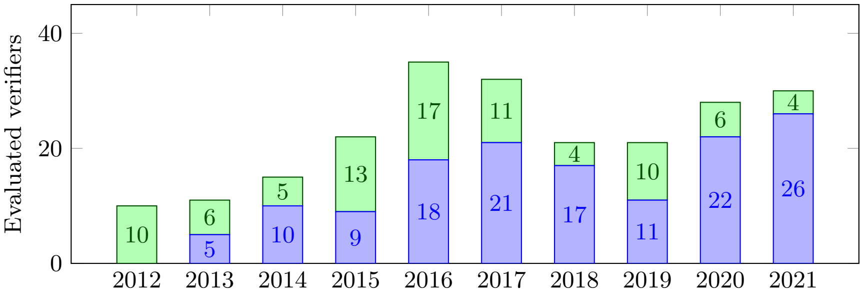

Fig. 5: Number of evaluated verifiers for each year (first-time participants on top)

Table 11 shows the confirmed versus unconfirmed results: the first column lists the verifiers of category *C-Overall*, the three columns for result TRUE reports the total, confirmed, and unconfirmed number of verification tasks for which the verifier answered with TRUE, respectively, and the three columns for result FALSE reports the total, confirmed, and unconfirmed number of verification tasks for which the verifier answered with FALSE, respectively. More information (for all verifiers) is given in the detailed tables on the competition web site and in the results artifact; all verification witnesses are also contained in the witnesses artifact (see Table 4). The verifiers 2LS and UKOJAK are the winners in terms of confirmed results for expected results TRUE and FALSE, respectively. The overall interpretation is similar to SV-COMP 2020 [17].

## 5 Conclusion

The 10th edition of the Competition on Software Verification (SV-COMP 2021) had 30 participating verification systems from 11 countries (see Fig. 5 for the participation numbers and Table 5 for the details). The competition does not only execute the verifiers and collect results, but also validates the verification results using verification witnesses. We used six independent validators to check the results and a witness linter to check if the verification witnesses are syntactically valid (Table 1). The number of verification tasks was increased to 15 201 in the C category and to 473 in the Java category. The high quality standards of the TACAS conference, in particular with respect to the important principles of fairness, community support, and transparency are ensured by a competition jury in which each participating team had a member. The results of our comparative evaluation provide a broad overview of the state of the art in automatic software verification. SV-COMP is instrumental in developing more reliable tools, as well as identifying and propagating successful techniques for software verification.

**Data Availability Statement.** The verification tasks and results of the competition are published at Zenodo, as described in Table 4. All components and data that are necessary for reproducing the competition are available in public version repositories, as specified in Table 3. Furthermore, the results are presented online on the competition web site for easy access: https://sv-comp.sosy-lab.org/2021/results/.
